# The History of Medicine and Surgery in Georgia

**Published:** 1894-12

**Authors:** Luther B. Grandy

**Affiliations:** Atlanta, Ga.


### THE HISTORY OF MEDICINE AND SURGERY IN GEORGIA.*

BY LUTHER B. GRANDY, M.D.,

ATLANTA, GA.

Dr. W. C. Daniell, of Savannah, was one of our most prominent and successful practitioners of this period. He also had given much study to the fevers of his section, and he embodied the results of many years' observation in a monograph entitled "The Autumnal Fevers in Savannah," published in 1826. This paper, though not in harmony with prevailing views among the profession, was regarded as an important contribution to the scanty medical literature of the day. The treatment of fractures of the thigh by the weight and pulley was first used in this country by Dr. Daniell. His first case occurred in 1819. To obtain continuous extension he folded a silk handkerchief securely around the patient's ankle and tied the ends across the sole of the foot. A cord and weight were then attached, and passed over a roller at the foot of the bed. A second case was similarly treated in 1824. The roller in this case was replaced by a pulley. Dr. Daniell described his cases in the *American Journal of Medical Sciences* for August, 1829†. His paper at once attracted the attention of Dr. Milton Antony, of Augusta, who "determined on adopting the method in the first case that should occur." In the *Southern Medical and Surgical Journal*, October, 1836, Dr. Antony reports five cases, "all of which had been treated on the plan herein detailed, and with like success." One of these cases was a man, aged forty-six. Fracture in upper third of thigh. "A

*Continued from page 465, October number.

†About 1831 Dr. Swift, of Pennsylvania, introduced adhesive plaster as a substitute for the silk handkerchief.

short roller bandage was passed around the ankle and bottom of the foot, where a string was attached, which, passing over the foot of the bed suspended a piece of brick weighing about two and a half pounds." Short splints were also applied about the point of fracture. Patient was discharged cured in about six weeks. Dr. Antony dispensed with the pulley of Dr. Daniell, merely allowing the cord to suspend from the end of the mattress or table. Dr. Antony's opinion of this method was that "it establishes, in the most satisfactory manner, the propriety of a plan of management at once calculated to insure the best success with the simplest apparatus and the least distress." It will thus be seen that many years before the so-called "Buck's Extension Apparatus" was introduced into the New York Hospital in 1852, every mechanical principle of that arrangement had been used and described by the above named surgeons. The general teaching, therefore, that this method of treating fractures of the thigh was originally suggested by Dr. Gurdon Buck, is wholly incorrect.

For the first thirty years of the century our chief point of medical interest was Savannah. For the next thirty years it was Augusta. The supremacy of this city in this respect was to a great extent due to the fact that it was the home of the Medical College of Georgia, and of *The Southern Medical and Surgical Journal.* In the opinion of the most prominent Augusta physicians the time had come for the establishment of a medical college farther south. Our young men were going North for their medical education. In 1825 Dr. Milton Antony applied to the legislature in Milledgeville for a charter for a "Medical Academy." The "Academy" was to be only a preparatory school to Northern colleges. It went into operation with three professors and a promising class. Its name was shortly afterwards changed to "Institute," and it was permitted to confer the degree of Bachelor of Medicine. In 1830 Dr. Antony and his friends still further elevated the character of the institution by obtaining a charter for the "Medical College of Georgia," with full power to teach medicine and surgery and confer degrees. Thus organized, the faculty consisted of Dr. L. A. Dugas, Professor of Anatomy; Dr. Jos. A. Eve, Materia Medica and Therapeutics; Dr. L. D. Ford, Chemistry and Pharmacy; Dr. Milton Antony,

Obstetrics and Diseases of Children; Dr. Paul F. Eve, Surgery; and Dr. John Dent, Practice of Medicine.

From its organization the Medical College of Georgia took a position in favor of the highest medical education which was offered at that time. The course of instruction extended over six months, and two courses were necessary for graduation. Even at that remote day some members of the faculty advocated "four years attendance upon lectures as necessary to render a candidate eligible to the doctorate." In May, 1835, the college, through Dr. L. D. Ford, Dean, addressed a circular letter to all the colleges in the country, suggesting a convention of representatives from the different colleges to be held in Washington, "for the purpose of establishing an uniform system of requirements for graduation; of regulating the courses of professional study; the extent of previous education; and counselling generally about the means of rendering our institutions most successful in diffusing the benefits of medical education." Nothing came of this excellent suggestion. Only a few colleges favored it. Others either made no reply, or declined co-operation on the ground that "the time had not yet come when a prolongation of the term is necessary." There were then fifteen medical schools in the United States. In some the course lasted five months; in others, only four. Failing in the above suggestion, the Medical College of Georgia, with its six-months course, saw that it could not compete with the short-term institutions; so in 1838 it was compelled reluctantly to reduce its own term to four months. A member of the faculty says apologetically: "If there be error, therefore, in the adoption of four months as the term of annual instruction, the blame is attributable to the retention of the short course by the other colleges."

The *Southern Medical and Surgical Journal* was the child of the college. It was organized by Dr. Antony, and the first number issued, June, 1836. It was the first medical journal to be established in the South. There were about half a dozen others in the country. The introduction to the new journal said: "The profession at the South have long regarded and anticipated, as a most desirable object, the establishment of a journal that should collect and preserve the valuable discoveries and improvements of Southern practitioners relative to the nature and treatment of diseases incident to Southern climates." The journal rapidly sprang to first place among the medical periodicals of the day. The faculty of the college made it the special object of their pride and care. It was the principal medium of publication of their own medical papers. It was ably edited, and among its pages are to be found scientific papers of the highest order from the most prominent medical men in the South.

The medical act of 1825, regulating the licensing of physicians to practice in the State had now been in operation several years. We are not informed in detail as to the workings of the board of examiners, but it is in evidence that they did good service. We have it on the authority of a contemporaneous writer* that previous to the passage of the above act incompetent physicians and those who ignorantly followed the vagaries of Thomsonianism were the rule, and intelligent, educated practitioners the exception, particularly in the interior of the State. However, "by the wholesome operation of this law during a short lifetime of only ten years, quackery was scouted out, and real science so pursued that no section of the country where there was any considerable population was without a practitioner of true science." Still, those who practiced the Thomsonian heresy were to be found in every part of the State. If we are to judge of the character of these parties and their practices by the ephithets which were applied to them by some of their contemporaries in the regular profession, the status of the botanic school must have been at a very low ebb. Dr. Delony, of Talbotton, refers to them as the "base charlatans who impose upon the credulity, destroy the lives, and rob the pockets of a too unwitting community." Dr. Antony said that the "degrading stain of Thomsonianism" was a "disgrace to the American character," and he regarded it as "the most stupendous system of quackery and the most insulting offering ever tendered to the understanding of a free and enlightened people." The act of 1825 had certainly furnished some protection against the incursions of these ignorant and unscrupulous practitioners. They themselves regarded this legislation as oppressive, and petitioned the legislature

*_Southern Medical and Surgical Journal_, November, 1837.

of 1836 for its repeal. Indeed, this petition even found favor among the regular profession. Some heartily advocated it. They "believed that the sure and more speedy method of allowing them (the botanic doctors) to sink into the neglect and contempt to which they are destined would be to place them upon equal ground with physicians, when the community could become convinced of the absurdity of their theory and the destructiveness of their practice." So, a new act was passed, repealing certain sections of the original act, "so far as they subject to fine or imprisonment persons practicing medicine consisting of vegetable and animal substances, caloric, etc., under the name and style of botanic physicians." Thus the law was emasculated, deprived of the very portions which made it useful and effective. A writer of the period says that this act of the legislature "legalized at one swoop a legion of impostors, an instance of retrograde legislation perhaps never equalled in the United States." The matter was again agitated and the original act was revived in 1839.

Frequent reference is made above to Dr. Milton Antony, of Augusta, possibly the most distinguished of our practitioners of this period. Circumstances had deprived him of the advantages of a completed education. He had attended only one course of medical lectures (in Philadelphia). But he possessessed both ambition and application, and with these he set about to prepare himself thoroughly for his professional work. He was a man of high ideals and noble purposes. With him medicine was almost a passion, and the elevation of the profession in Georgia was the end toward which he devoted seventeen years of unselfish and unremitting labor. One surgical operation which he performed in his early career deserves some notice. It was probably the first operation upon the lung, and even in the light of present-day surgical facilities, we must regard it as judiciously conceived and boldly executed. The case was reported by Dr. Antony in the Philadelphia *Medical Journal*, 1823. The patient presented in March, and concluded to open the chest. Beginning at the sternum he made a long incision over the fifth interspace down to the lower end of the tumor. A copious discharge of old blood and lymph coagula followed. The sixth rib was necrosed and its anterior half was removed in small fragments. Similarly with the fifth rib. Having taken away the grumous and coagulated matter within the thorax, Dr. Antony proceeded to examine the lung. A large part of the lung tissue was pulpy and disorganized, and the right lobe extensively destroyed. All such parts within reach of the fingers were removed without violence or the risk of producing hemorrhage. The wound was then cleansed and covered with a plaster, and a roller applied so tightly as to prevent motion of the ribs. The patient was not cured, but much benefited by the operation. He died of measles one year and a half later.

In 1825 it was Dr. Antony and a few others who secured the medical act providing for a board of examiners. Dr. Antony was made first president of the board. His establishment of the medical college and the medical journal have already been mentioned. He was severe in denouncing the legislature of 1836 for repealing the essential parts of the act of 1825, and through the efforts of himself and others the original act was re-established.

Dr. Antony was highly esteemed as a learned and scientific physician, and he achieved a wide reputation. The honorary degree of Doctor of Medicine was bestowed upon him by two distinguished universities. Readers of his journal often sought his opinion on medical questions through its pages. This journal contained about all his contributions to medical literature. We find papers of his entitled "Menstrual Irregularities," "Remarks on Puerperal Convulsions," "The Causes of Abortions," "The Treatment of Fracture of the Femur by Weight and Fulcrum," "Medical Electricity," "Maternal Impressions," "Remarks on the Sulphate of Quinine," and "Contributions to Obstetrics," the last being a report of the most interesting and instructive cases gathered from his obstetric records. Dr. Antony died during the fatal epidemic of 1839, "A martyr to humanity and the duties of his profession. He passed from the cure of the sick to the sleep of the just, amid the tears and blessings of the poor."*

(*To be continued.*)

*Inscription on memorial tablet in the college at Augusta.

